# An Event-Related Potential Investigation of the Effects of Age on Alerting, Orienting, and Executive Function

**DOI:** 10.3389/fnagi.2016.00099

**Published:** 2016-05-09

**Authors:** David A. S. Kaufman, Christopher N. Sozda, Vonetta M. Dotson, William M. Perlstein

**Affiliations:** ^1^Department of Psychology, Saint Louis UniversitySt. Louis, MO, USA; ^2^Department of Clinical and Health Psychology, University of FloridaGainesville, FL, USA; ^3^Malcom Randall Veterans Administration Medical CenterGainesville, FL, USA; ^4^McKnight Brain Institute, University of FloridaGainesville, FL, USA; ^5^Departments of Psychiatry, Saint Louis UniversitySt. Louis, MO, USA; ^6^VA RR&D Brain Rehabilitation Research Center of Excellence, Malcom Randall Veterans Administration Medical CenterGainesville, FL, USA

**Keywords:** ANT, attentional networks, alerting, N1, aging, ERPs

## Abstract

The present study compared young and older adults on behavioral and neural correlates of three attentional networks (alerting, orienting, and executive control). Nineteen young and 16 older neurologically-healthy adults completed the Attention Network Test (ANT) while behavioral data (reaction time and error rates) and 64-channel event-related potentials (ERPs) were acquired. Significant age-related RT differences were observed across all three networks; however, after controlling for generalized slowing, only the alerting network remained significantly reduced in older compared with young adults. ERP data revealed that alerting cues led to enhanced posterior N1 responses for subsequent attentional targets in young adults, but this effect was weakened in older adults. As a result, it appears that older adults did not benefit fully from alerting cues, and their lack of subsequent attentional enhancements may compromise their ability to be as responsive and flexible as their younger counterparts. N1 alerting deficits were associated with several key neuropsychological tests of attention that were difficult for older adults. Orienting and executive attention networks were largely similar between groups. Taken together, older adults demonstrated behavioral and neural alterations in alerting, however, they appeared to compensate for this reduction, as they did not significantly differ in their abilities to use spatially informative cues to aid performance (e.g., orienting), or successfully resolve response conflict (e.g., executive control). These results have important implications for understanding the mechanisms of age-related changes in attentional networks.

## Introduction

Intact attentional processes are vital for goal-directed behavior, as they guide the allocation of cognitive resources in accordance with changing environmental demands. Research from the past several decades has proposed that attention is comprised of dissociable yet interrelated anatomical and functional neural networks responsible for alerting, orienting, and executive control (Posner and Peterson, 1990; Posner and Fan, 2004). The alerting system helps us to achieve and sustain an alert state (Fernandez-Duque and Posner, 1997), which is thought to facilitate response preparation via enhanced early attentional processing. The orienting system functions to select information from sensory input, and through spatial cuing, can be manipulated to covertly direct attention (Posner, 1980; Fan et al., 2002). Lastly, executive control of attention encompasses higher-order cognitive processes needed to resolve conflict associated with the need to override a prepotent, but contextually inappropriate response (Fan et al., 2002). These executive operations are particularly crucial when situations are novel or difficult, and require response monitoring or inhibition of strong prepotent response tendencies (Fan and Posner, 2004).

In order to probe these different attentional networks, Fan et al. (2002) developed a single-trial computerized task known as the Attention Network Test (ANT). The ANT combines aspects of Posner and Peterson (1990) spatial cuing task with features of the Eriksen Flanker task (Eriksen and Eriksen, 1974), such that target flankers are spatially and/or temporally cued. Moreover, the ANT has been used to characterize the three attentional networks in studies employing behavioral (e.g., Fan et al., 2002), electrophysiological (e.g., Fan et al., 2007; Neuhaus et al., 2007), and hemodynamic (e.g., Fan et al., 2005; Posner et al., 2006) methodologies. Despite the popularity of this task, cautions have been raised about its psychometric properties. A large meta-analysis found poor split-half reliabilities within alerting and orienting network effects, along with significant inter-network correlations between the various networks (MacLeod et al., 2010). Accordingly, these attentional networks may not be as reliable or independent as initially thought, and it is important for researchers to document these psychometric properties when reporting individual study findings.

Several studies have used the ANT to examine how attentional networks are affected by aging processes; however, findings have been mixed. After controlling for the effects of generalized cognitive slowing, older adults have demonstrated weakened alerting effects, relative to younger adults (Jennings et al., 2007; Gamboz et al., 2010). In contrast, when longer cue presentation was employed, Fernandez-Duque and Black (2006) found a significantly larger alerting effect in older compared to young adults. Recent findings did not reveal significantly differing orienting effects between young and older adults (Jennings et al., 2007), a result consistent with prior literature that spatial cueing benefits older adults as much as young adults (Hartley et al., 1990; Folk and Hoyer, 1992; Greenwood et al., 1993). Regarding executive control, no significant conflict-related effects have been observed in several studies comparing older and young adults (Fernandez-Duque and Black, 2006; Jennings et al., 2007). In contrast, a more recent study suggests age-related changes may occur in the executive control network (Zhou et al., 2011). Taken together, it remains unclear whether age-related attentional declines may be confounded by cognitive slowing (Verhaeghen and De Meersman, 1998; Verhaeghen and Cerella, 2002), or other cognitive disruptions beyond slowed speed of information processing (Hartley, 1993).

Electrophysiological methodologies have provided further insight about the underlying neural mechanisms of attentional networks. In particular, studies of attentional processes (e.g., Fan et al., 2007; Neuhaus et al., 2007, 2010) have employed scalp-recorded brain event-related potentials (ERPs), which provide unmatched temporal sensitivity for examining neural correlates. Despite recent electrophysiological investigations, the alerting network has received the least focus of the three attentional networks in the literature. With the goal of examining effects of alerting cues on subsequent attentional processing, one study reported significant increases in N1 amplitude evoked by targets that followed alerting cues (Neuhaus et al., 2010). This posterior N1 component is believed to reflect early attentional facilitation of target processing, which is enhanced by valid cues that reliably predict target location (Hillyard et al., 1998).

With respect to orienting of visual attention, researchers have also observed increased amplitude of posterior negativity beginning approximately 100 ms following validly cued target stimuli (Harter et al., 1989; Hopf and Mangun, 2000; Nobre et al., 2000; Talsma et al., 2005). These results have been replicated using the ANT (Neuhaus et al., 2010), and are presumed to reflect the successful shift of selective visual attention from one stimuli to another in response to validly cued stimuli (Mangun, 1995; Hillyard et al., 1998). Neuroimaging findings suggest activation within a dorsal fronto-parietal network is strongly associated with orienting of attention (e.g., Fan et al., 2005).

Executive aspects of attention can be measured many different ways, with researchers typically targeting higher-order cognitive processes involved in conflict processing, inhibition, or decision-making. One method that has been frequently studied is the flanker task, in which compatibility of stimuli surrounding target stimuli is manipulated to vary the level of response inhibition needed. ERP investigations using the ANT in healthy young adults have revealed attenuation of posterior positivity following incongruent target stimuli (Neuhaus et al., 2007, 2010). Although the P300 is commonly observed in paradigms that probe attentional resource allocation (see Polich, 2007), it is also susceptible to conflict effects. For example, incongruent Stroop stimuli have been associated with negative deflections occurring during the P300 response time window (approximately 450 ms following stimulus onset). Although these conflict-sensitive N450 components have been shown to exhibit a more frontocentral scalp distribution (van Veen and Carter, 2002; Larson et al., 2009), cognitive/response conflict also appears to affect the more parietal-maximal P300. Consistent with the literature on the N450 component, these negative deflections during the P300 likely reflect anterior cingulate cortex (ACC) mediated conflict-resolution associated with the inhibition of a strong pre-potent response tendency (Fan et al., 2005, 2007; Neuhaus et al., 2010).

While the above results demonstrate the utility of the ANT to elucidate the presence of the three neural attentional networks, no published studies to date have examined the neural correlates of the ANT in *older* adults. Additionally, although a growing body of behavioral literature suggests age-related impairments in each attentional network, findings are mixed and often confounded by generalized slowing in older participants. Consequently, the primary aim of the current study was to use ERPs to supplement behavioral data to determine whether significant between-group neural differences are observed in the presence-and/or absence of significant behavioral deficits, as well as if neural correlates are more closely associated to behavioral findings that are corrected for generalized slowing than when uncorrected. Guided by prior electrophysiological findings of Neuhaus et al. (2007, 2010), ERP components of interest included the posterior N1 (for alerting and orienting networks), and parietal P300 (executive control network) components, which are presumed to reflect phasic alerting, orienting of attention, and processing of response conflict (executive control), respectively.

## Materials and Methods

### Participants

Participants included 19 young (12 females, 7 males; mean age 22.9 ± 4.0 years) and 16 older (8 females, 8 males; mean age 64.8 ± 8.0 years) adults recruited from the community through local advertisements. All participants were right-handed, native-English speakers, and had normal or corrected-to-normal vision. Groups were matched for gender, χ2_(1)_ = 0.62, *p*s > 0.43. Although older adults reported a higher mean level of educational attainment than young adults, *t*_(33)_ = −2.70, *p* < 0.02, education did not correlate with ANT RT, error-rates, or ERP amplitudes collapsed across conditions (*p*’s > 0.052). Education was significantly correlated with Stroop performance, *r*_(34)_ = 0.49, *p* < 0.003, however, this measure is typically only corrected for age and not education in standard clinical practice. Exclusion criteria included presence of psychiatric illness, learning disability, probable dementia or global cognitive impairment (as determined by a MMSE score <25), neurological disorders, and motor difficulties that would interfere with task performance. All participants provided written informed consent in accordance with procedures established by the University of Florida Health Science Center Institutional Review Board, and received course credit or financial compensation for their study participation.

### Procedures

#### Cognitive and Emotional Assessment

Participants completed the following neuropsychological tests: Mini-Mental State Exam (MMSE; Folstein et al., 1975), Trail Making Test A and B (TMT; Reitan and Wolfson, 1995), Digit-Symbol Coding from the Wechsler Adult Intelligence Scale, 3rd Edition (WAIS-III; Wechsler, 1997), and Stroop Color and Word Test (ST; Golden, 1978). The Beck Depression Inventory-Second Edition (BDI-II; Beck, 1996), Geriatric Depression Scale (GDS; Yesavage et al., 1983), State-Trait Anxiety Inventory (STAI; Speilberger et al., 1983), and modified Apathy Scale (AS; Starkstein et al., 1992), were used to measure symptoms of depression, anxiety, and apathy, respectively. Demographic and neuropsychological characteristics of participants are presented in Table 1.

**Table 1:** **Demographic and neuropsychological data for young and older participant groups**.

	Young Adults		Older Adults			Analysis	
	Mean	SD	Mean	SD	*t*	*p*	Cohen’s *d*
Age (years)	22.95	4.0	64.81	8.0	−20.02	<0.001	6.81
Educational level (years)	15.05	1.2	17.13	3.1	−2.70	0.011	0.92
BDI-II score	3.05	3.2	2.88	3.0	0.16	ns	0.05
GDS score	1.79	2.4	0.94	1.3	1.26	ns	0.43
STAI—State score	26.79	5.2	27.56	7.2	−0.37	ns	0.12
STAI –Trait score	31.10	7.3	28.50	5.2	1.20	ns	0.40
Apathy Scale score	8.10	4.8	8.81	4.6	−0.47	ns	0.15
MMSE Score	28.95	1.0	28.69	1.3	0.69	ns	0.23
Stroop Test—Word-reading	99.26	12.3	107.06	17.4	−1.55	ns	0.53
Stroop Test—Color-naming	74.37	9.0	77.50	13.8	−0.81	ns	0.27
Stroop Test—Color-word	45.31	10.1	46.19	13.4	−0.22	ns	0.08
Stroop Test—Interference	2.99	8.8	1.51	9.3	0.48	ns	0.16
Digit symbol coding (# correct)	89.89	13.1	72.87	11.6	3.95	<0.001	1.38
TMT—Part A (sec)	22.16	4.1	31.06	9.8	−3.61	0.001	1.22
TMT—Part B (sec)	50.53	18.2	67.81	27.9	−2.20	0.035	0.75
TMT—Part B minus Part A (sec)	28.37	16.9	36.75	22.2	−1.27	ns	0.43

*BDI-II, Beck Depression Inventory—Second Edition; GDS, Geriatric Depression Scale; STAI, State-Trait Anxiety Inventory; MMSE, Mini-Mental State Exam; TMT, Trail Making Test*.

#### Cognitive Task and Stimuli

**Figure 1:**
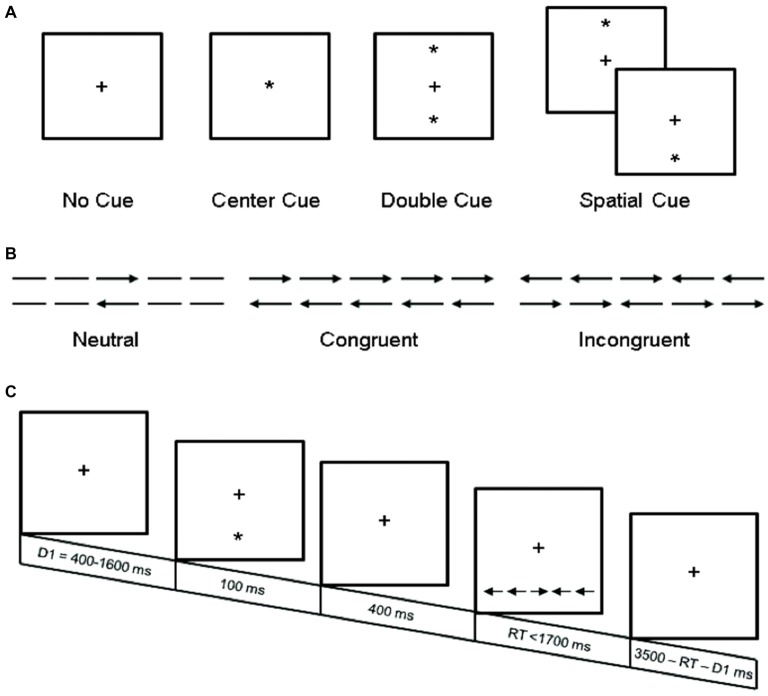
**Experimental procedure. (A)** The four cue conditions, in which the asterisk cue (*) provides information about the presence (center and double cue) or location (spatial cue) of the upcoming target stimuli; **(B)** The six target stimuli used in the present experiment; and **(C)** An example trial (spatial cue—incongruent target stimuli). Adapted from Fan et al. (2002).

Participants completed a computerized experimental task identical to the procedure used by Fan et al. (2002), as depicted in Figure 1. Stimuli were programmed and presented using E-Prime (v.1.0, Psychology Software Tools, Inc.,). Briefly, individuals were instructed to focus on a centrally located fixation cross throughout the procedure, and to determine as rapidly and accurately as possible whether the target probe, a central arrow, located above or below central fixation pointed left or right. In addition to varying target locations, on 75% of trials the target probe was preceded by different types of cues, and on 67% of trials the target probe was accompanied by congruent or incongruent flankers (which were equally frequent). The task utilized two target locations (above or below central fixation), two target directions (left or right), four cue conditions (no-cue, center-cue, double-cue and spatial-cue), and three flanker conditions (congruent, incongruent, or neutral), yielding 48 different types of trials. Each trial lasted ~4000 ms and consisted of five events: (1) random variable pre-cue fixation (400–1600 ms); (2) warning cue presentation (100 ms); (3) post-cue fixation (400 ms); (4) target and flanker presentation (self-terminating up to 1700 ms); and (5) post-target fixation (3500 ms minus duration of pre-cue fixation minus RT). Participants completed a 24-trial practice block, followed by 3-experimental blocks of 96 pseudo-random trials (2 target locations × 2 target directions × 2 repetitions × 3 flanker conditions × 4 cue conditions). Accuracy and RT feedback were provided to participants only during the practice block.

#### Electrophysiological Data Acquisition and Reduction

Electroencephalogram (EEG) was recorded from 64 scalp sites using a geodesic sensor net and Electrical Geodesics, Inc., (EGI; Eugene, Oregon) amplifier system (20 K gain, nominal bandpass = 0.10–100 Hz). Reported electrode sites have been renamed from the 64-channel geodesic sensor net to conform to the international 10–10 positions. Electrode placements enabled recording vertical and horizontal eye movements reflecting electro-oculographic (EOG) activity. EEG was digitized continuously at 250 Hz with a 16-bit analog-to-digital converter and referenced to Cz. A right posterior electrode served as common ground, and electrode impedances were maintained below 50 kΩ, consistent with manufacturer recommendations. Following recording, EEG data were adjusted for movement, electromyographic muscle artifact, electro-ocular eye movement, and blink artifacts using spatial filtering methods implemented through Brain Electric Source Analysis (BESA v5.1; Scherg, 1990). Single-trial EEG epochs were excluded from the averaging using threshold criteria that maximized the number of trials accepted from each individual, following the recommendations of Luck (2005). The average voltage threshold used for excluding trials was 113.86 μV (*SD* = 16.89), and voltage threshold did not significantly differ between groups, *t*_(33)_ = 1.46, *p* = 0.16. Point-to-point transitions were not allowed to exceed 75 μV. Individual-subject per condition EEG epochs were separately extracted and averaged across trials from the continuous EEG recording, with epochs lasting 1600 ms (300 ms pre-cue to 800 ms post-probe including 400 ms cue-offset–probe-onset interval). Cue-locked target ERP components were examined to observe the sustained effects of cue on subsequent target processing, in replication of methods used previously (Neuhaus et al., 2010). All averaged ERP epochs were digitally filtered at 30 Hz low-pass and baseline-corrected using respective pre-stimulus windows. Peak voltage values for target-related N1 amplitude were measured bilaterally over posterior parietal scalp sites (10–10 system equivalents = P9, PO7, PO8, P10) between 176–216 ms post-target onset. Because of group-related latency differences apparent in P300 grand-average waveforms, ERPs were scored such that mean P300 amplitude was measured from 380–410 ms for young adults and 500–530 ms for older adults at central-posterior electrode sites (10–10 equivalents = CPz, Pz, and POz), averaged 30-ms symmetrically about the peak. The scoring windows for each group were determined by identifying the maximum peak amplitude of P300 within grand-averaged waveforms for each age group.

### Data Analyses

#### Behavioral Performance Data

Statistical analyses were carried out with JMP 7.0.2 (SAS Institute Inc., Cary, NC, USA). Attention network effects and neuropsychological variables were analyzed using one-way mixed-model restricted maximum likelihood analyses of variance (REML ANOVAs; Wolfinger et al., 1994). Median correct-trial RT (Ratcliff, 1993) and arcsine transformed commission-error rates, excluding non-responses (Neter et al., 1985), were analyzed separately using 2-Group (young adults, older adults) × 3-Flanker type (incongruent, neutral, congruent) × 4-Cue type (no, spatial, double, and center) mixed-model REML ANOVAs. In order to follow up on these ANOVA results, attention network effects were calculated using the following cognitive subtractions: *alerting effect* = no-cue RT minus double-cue RT; *orienting effect* = center-cue RT minus spatial cue RT; *executive control (conflict) effect* = incongruent RT minus congruent RT. For ANOVA factors that differed by group, independent *t*-tests were used to compare attentional network effects between young and older adults. Additionally, to examine whether condition-related RT effects were artificially created due to generalized slowing experienced by older adults, we calculated a *z*-score transformation of each participant’s RT by taking the mean over all conditions for a given individual, subtracting his/her condition mean from the overall mean, and dividing by the overall standard deviation across the overall mean. This method has been proven effective in identifying age-related cognitive effects independent of global slowing (Bush et al., 1993; Faust et al., 1999; Jennings et al., 2007). Finally, split-half reliabilities for each network effect were calculated by correlating data from the first and second halves of trials within each network.

#### ERP Data

In a manner parallel to the behavioral data, ERP activity from attention networks was analyzed using REML ANOVAs, which included the following factors: Group × Channel (N1 analyses = 10–10 equivalents P9, PO7, PO8, P10; P3 analyses = 10–10 equivalents CPz, Pz, and POz), × Condition (either double cue and no cue, spatial cue and center cue, or congruent probe and incongruent probe, depending on the attention network being analyzed). Selection of electrode sites for analyses of electrophysiological data was based on evaluation of the scalp-distribution maps in which N1 and P300 amplitudes were maximal. Interaction effects were decomposed using least-square means contrasts; and Cohen’s-*d* effect sizes (Cohen, 1988) were calculated using pooled standard deviations for group and/or condition-related effects. ANOVAs were then followed up with the calculation of attention network effects, using the same paired subtractions that were used to generate the behavioral attention network effects. ERP network effects were derived using the following cognitive comparisons: *alerting N1 effect* = double-cue amplitude − no-cue amplitude; *orienting N1 effect* = spatial cue amplitude − center-cue amplitude; *executive control P3 (“conflict”) effect* = congruent amplitude − incongruent amplitude. Group differences on these ERP network effects were analyzed using independent *t*-tests. Mean amplitudes for ERP network effect were then correlated with neuropsychological measures that differentiated young and older adults.

## Results

### ANT Behavioral Performance

A Group × Flanker type × Cue type ANOVA on correct-trial median RTs revealed a significant main effect of group, *F*_(1,33)_ = 38.75, *p* < 0.0001, reflecting the expected pattern of generalized slowing in older adult participants. As expected, a significant main effect of flanker type, *F*_(2,363)_ = 832.73, *p* < 0.0001, was also observed, reflecting increased slowing when faced with incongruent flankers. Participants responded more quickly as cue types became more informative (no cue RT < center cue RT < double cue RT < spatial cue RT), as evidence by a significant main effect of cue type, *F*_(3,363)_ = 137.96, *p* < 0.0001. Additionally, a significant Group × Flanker type interaction, *F*_(2,363)_ = 4.59, *p* < 0.02, indicated that older adult participants responded significantly more slowly compared to neutral trials for both congruent and incongruent stimuli, whereas young adults only displayed slowing compared to neutral stimuli for incongruent target conditions. A Group × Cue-type interaction, *F*_(3,363)_ = 5.20, *p* < 0.002, indicated that older adults did not benefit from center cues as much as young adults. Flanker type × Cue type, *F*_(6,363)_ = 1.47, *p* = 0.19, and Group × Flanker type × Cue type, *F*_(6,363)_ = 1.54, *p* = 0.16, interactions were not significant.

Attention network difference scores revealed significant RT differences between groups for alerting (Young = 46 ± 25 ms; Older = 18 ± 32 ms), *F*_(1,33)_ = 7.69, *p* < 0.01, orienting (Young = 43 ± 27 ms; Older = 87 ± 32 ms), *F*_(1,33)_ = 19.81, *p* < 0.0001, and executive control (Young = 117 ± 22 ms; Older = 135 ± 29 ms), *F*_(1,33)_ = 4.32, *p* < 0.05, effects. However, after normalization of RT data to account for generalized slowing experienced by older adults using *z*-score transformations, significant group differences remained only for the alerting effect, *F*_(1,33)_ = 8.02, *p* < 0.01. In other words, young adults responded faster following alerting cues, but older adults did not—even after controlling for group differences in overall speed. Group differences in the orienting effect, *F*_(1,33)_ = 0.39, *p*s > 0.53, and conflict effect *F*_(1,33)_ = 2.39, *p*s > 0.13, were no longer significant following normalization to control for generalized slowing. Mean attention network effects, *z*-score transformed attention network effects, and correct-trial median RT data for both groups as a function of flanker type and cue type are summarized in Table 2.

**Table 2:** **Mean (± SD) attention network effects (RT), *Z*-score transformations for attention network effects, and arcsine transformed error rates as a function of flanker-type and group**.

	Young Adults		Older Adults			Analysis	
	Mean	SD	Mean	SD	*t*	*p*	Cohen’s *d*
Mean RT (ms)	485.16	44.9	646.44	109.6	−5.87	<0.001	1.99
Alerting effect (ms)	46.29	24.9	17.78	32.1	2.77	0.009	1.00
Orienting effect (ms)	43.08	27.0	87.41	31.9	−4.45	<0.001	1.51
Conflict effect (ms)	117.29	22.3	135.41	29.2	−2.08	0.050	0.71
*Z*-score RT transformations
Alerting effect	0.68	0.34	0.27	0.50	2.83	0.008	0.98
Orienting effect	0.65	0.40	0.76	0.62	−0.63	0.540	0.21
Conflict effect	1.64	0.36	1.39	0.58	1.54	0.140	0.53
Mean error rates (%)	6.20	12.4	5.40	10.5	−0.73	ns	0.07
Congruent	1.90	6.0	2.40	7.0	0.46	ns	0.08
Incongruent	13.70	16.6	9.60	13.3	−1.59	ns	0.27
Neutral	3.00	8.1	4.20	8.8	0.79	ns	0.14

*Alerting, no cue (RT) minus double cue (RT); Orienting, center cue (RT) minus spatial cue (RT); Conflict, incongruent (RT) minus congruent (RT)*.

Split-half reliabilities on RT data revealed that the conflict effect had the highest internal consistency (*r*_(33)_ = 0.41, *p* < 0.05), followed by alerting (*r*_(33)_ = 0.37, *p* < 0.05) and orienting (*r*_(33)_ = 0.29, *p* = 0.09) effects. When analyzed separately by group, network reliabilities were much lower for older adults than young. Young adults had significant split-half reliabilities for the conflict (*r*_(17)_ = 0.54, *p* < 0.05) and alerting (*r*_(17)_ = 0.48, *p* < 0.05) networks, while older adults had no significant reliabilities within networks. Alerting and conflict effects were significantly correlated in younger adults (*r*_(17)_ = −0.50, *p* < 0.05), but no other cross network correlations were significant for task RT.

Regarding task performance accuracy, a Group × Flanker type × Cue type ANOVA revealed a significant main effect of flanker type, *F*_(2,363)_ = 36.56, *p* < 0.0001, reflecting the expected larger error-rates on incongruent than congruent trials. A significant main effect of cue type *F*_(3,363)_ = 4.26, *p* < 0.006, was also observed, indicating greater accuracy to targets following spatial cues. The group main effect, *F*_(1,33)_ = 0.30, *p* = 0.59, and Group × Congruency interaction, *F*_(2,363)_ = 2.84, *p*s > 0.06, were not significant, suggesting equal task performance across groups regardless of task difficulty. Finally, a significant Group × Flanker type × Cue-type, *F*_(6, 363)_ = 2.17, *p* < 0.05, revealed that spatial cuing improved accuracy on incongruent trials for young, but not older adults. This finding reveals that conflict processing is improved following spatial cues for young adults only, and may suggest that older adults fail to benefit fully from spatial cueing when increased conflict is present. With regard to task accuracy, attention network difference scores did not differ as a function of group, which is likely attributable to the low error rates seen in both groups. Of additional note, a negative correlation was observed between alerting effect RT and alerting effect error rate for young adults (*r*_(17)_ = −0.61, *p* < 0.01), suggesting that they exhibited a speed-accuracy tradeoff when engaging the alerting network. This effect was not seen in older adults (*r*_(14)_ = −0.25, *p*s > 0.35).

### ERP Components

As shown in Table 3, the average number of trials included in ERPs did not differ significantly between groups for target N1 alerting and orienting components. However, older adults had more trials included in the target P300 conflict component relative to younger adults.

**Table 3:** **Mean (± SD) number of ERP segments comprising each component, as a function of group**.

	Young Adults		Older Adults			Analysis	
	Mean	SD	Mean	SD	*t*	*p*	Cohen’s *d*
ERP Component
Target N1 Alerting	43.82	10.24	49.66	9.87	−1.71	0.10	0.59
Target N1 Orienting	44.50	10.97	49.53	9.73	−1.42	0.16	0.49
Target P300 Conflict	57.95	13.04	68.63	11.62	−2.53	0.02	0.88

### Target N1 Alerting Effect (Double-cue vs. No-cue)

**Figure 2:**
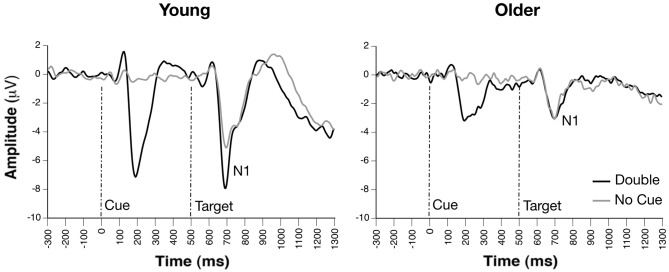
**Grand average event-related potential (ERP), waveforms of cue- and probe-locked double and no cue trials, revealing group differences on alerting effects on target-related N1 amplitudes (channel PO7)**.

Occipito-parietal waveforms illustrating grand average ERP waveforms for the alerting effect are shown in Figure 2. The Group × Condition × Channel ANOVA yielded significant main effects of group, *F*_(1,33)_ = 10.20, *p* = 0.0031, channel, *F*_(3,231)_ = 6.47, *p* = 0.0003, and condition, *F*_(1,231)_ = 8.96, *p* = 0.0031. A significant Group × Channel interaction, *F*_(3,231)_ = 4.04, *p* = 0.0079, indicated that voltages did not significantly differ between any channels for older adults, however, young adults demonstrated significantly greater voltage fluctuations between channels. A Group × Condition interaction, *F*_(1,231)_ = 15.07, *p* < 0.0001, revealed that young adults demonstrated larger N1 amplitude for double cues relative to no cue, *t*_(18)_ = 1.90, *p* = 0.001, *d* = 0.90, while older adults showed no difference between these two cue conditions, *t*_(15)_ = −0.25, *p*s > 0.54. Attention network difference scores calculated from peak amplitudes supported these ANOVA results with significant N1 alerting amplitude differences between groups, Young = −2.58 ± 2.62 μV; Older = 0.17 ± 1.35 μV, *t*_(33)_ = 3.99, *p* < 0.001, suggesting that young adults demonstrated a strong alerting effect on N1 amplitude, while older adults did not.

### Target N1 Orienting Effect (Spatial-cue vs. Center-cue)

**Figure 3:**
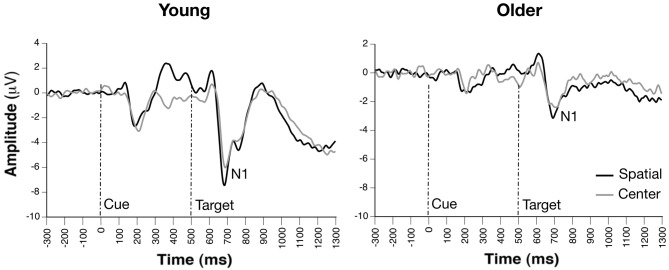
**Grand average ERP waveforms of cue- and probe-locked spatial and center cue trials, revealing similar orienting effects on target-related N1 amplitudes between groups (channel PO7)**.

Occipito-parietal waveforms illustrating grand average ERP waveforms for the orienting effect are shown in Figure 3. The Group × Condition × Channel ANOVA yielded significant main effects of group, *F*_(1,33)_ = 14.22, *p* = 0.0006, condition, *F*_(1, 231)_ = 8.35, *p* = 0.0042, and channel, *F*_(3,231)_ = 3.59, *p* < 0.015. The main effect of group reflected significantly smaller N1 amplitude in older compared to young adults, while increased amplitude to spatial vs. center cue was revealed in the main effect of condition. The effect of channel revealed greatest amplitude at PO7 and smallest amplitude at P10. No significant interactions were observed (*p*s > 0.09). Furthermore, attention network difference scores calculated from peak N1 orienting amplitudes revealed no group differences (*p* > 0.09).

### Target P300 Conflict Effect (Congruent vs. Incongruent)

**Figure 4:**
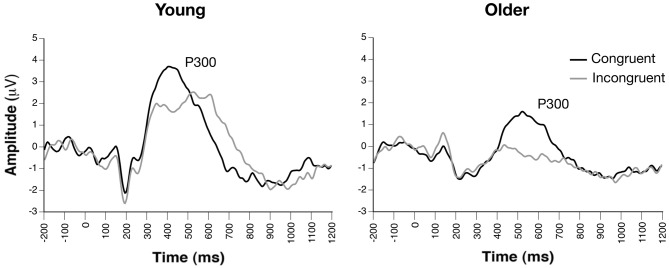
**Grand average ERP waveforms of target-locked congruent and incongruent trials, revealing similar conflict effects on target-related P300 amplitudes between groups (channel Pz)**.

Parietal waveforms illustrating conflict-related target processing are illustrated in Figure 4. The Group × Condition × Channel ANOVA yielded significant main effects of group, *F*_(1, 33)_ = 11.90, *p* = 0.0016, condition, *F*_(1, 165)_ = 39.32, *p* < 0.0001, and channel, *F*_(2, 165)_ = 4.02, *p* < 0.02. The main effect of group reflected significantly smaller P300 amplitude collapsed across target conditions in older compared to young adults, while reduced amplitude to incongruent vs. congruent cue was revealed in the main effect of condition. The effect of channel revealed greatest amplitude at CPz and smallest amplitude at POz. No significant interactions were observed (*p*s > 0.37). Additionally, attention network difference scores calculated from peak P300 conflict amplitudes revealed no group differences (*p* > 0.90).

### Relationships Between Behavioral and ERP Attention Network Effects

Mean N1 alerting amplitude taken from the channel with maximal amplitude (PO7) was not correlated with RT alerting effects (no cue − double cue), but N1 alerting amplitude was significantly correlated with performance on Digit Symbol Coding (*r*_(33)_ = −0.50, *p* < 0.01), Trails A (*r*_(33)_ = 0.52, *p* = 0.001), and Trails B (*r*_(33)_ = 0.42, *p* = 0.01). Mean N1 orienting amplitude (taken from PO7) was not correlated with RT orienting effects (center cue − spatial cue) or any other neuropsychological variables. Mean P300 conflict amplitude (taken from Pz) was not correlated with RT conflict effects (incongruent − congruent) or any other neuropsychological variables.

## Discussion

This study employed behavioral measures and high-density ERPs to probe age-related changes in the functioning of three attentional networks (alerting, orienting, and executive control). After controlling for effects of generalized slowing, only the alerting network remained significantly different in older compared with young adults, as suggested by limited facilitation of RT when provided with a double cue compared to no cue. With respect to ERP correlates of alerting, double cues significantly enhanced target-related N1 amplitude relative to the no-cue condition in young adults, consistent with prior findings of enhanced early attentional processing of targets following alerting cues (Neuhaus et al., 2010). While this N1 alerting effect was pronounced in younger adults, it was completely absent in older adults. These results support prior findings that early attentional processes reflected in the N1 are attenuated in older adults (Ford et al., 1995; Golub et al., 2001). Even more importantly, N1 alerting amplitude during the ANT was correlated with performance on three neuropsychological measures of controlled attention (i.e., Digit-Symbol Coding, Trails A and B) that differentiated older and younger adults. These findings reveal a unique vulnerability of the alerting network in aging, and suggest that compromised alerting in older adults is associated with broader difficulties in controlled attention.

While older adults demonstrated difficulty in using alerting cues to prepare for subsequent events, they were not impaired in their ability to use spatially informative cues to aid performance (orienting), or in their ability to successfully resolve response conflict (executive control). These results parallel the findings of other studies (e.g., Jennings et al., 2007; Gamboz et al., 2010) that also found age-related declines in the alerting network but intact functioning in other networks, after generalized slowing was taken into account. With regard to performance accuracy, both young and older participants committed significantly more errors during the more difficult incongruent flanker condition vs. neutral and congruent conditions. However, error rates did not significantly differ between groups, suggesting that older adults successfully inhibited strong prepotent response tendencies as well as their younger peers. Despite this, there were group differences in the relationship between error rate and response time for the alerting effect. While young adults adopted a strong speed-accuracy tradeoff when engaging the alerting network, older adults did not show this tendency. This finding is consistent with prior research on the speed-accuracy tradeoff in aging (Forstmann et al., 2011), which has suggested that older adults may have difficulty speeding up their response tendencies due to disrupted brain connections (particularly in corticostriatal pathways).

Regarding ERP correlates of visual orienting of attention, spatial cues were found to significantly enhance N1 amplitude vs. center cues in both young and older adult participants. Both groups appeared to engage neural processes underlying the orienting of attention and subsequent processing of target information equally well. These results also imply that valid cueing was beneficial and had a lasting effect on subsequent processing of target stimuli, a result consistent with other ERP examinations of the ANT demonstrating enhanced N1 during attentional orienting (Neuhaus et al., 2010), as well as studies of orienting of visual attention which found similar enhancements in N1-like posterior negativities following validly-cued target stimuli (Harter et al., 1989; Hopf and Mangun, 2000; Nobre et al., 2000; Talsma et al., 2005). Such results also suggest that although older adults may demonstrate less efficient response preparation as evidenced by reduced alerting, they are still able to shift attentional resources under the guidance of spatially informative cues.

Finally, examination of target-related conflict P300 revealed that both young and older adults showed significant and equivalent P300 amplitude reductions to incongruent vs. congruent target stimuli. Older adults did show reduced P300 amplitudes overall, as has been shown previously (e.g., Fjell and Walhovd, 2001); however, this group effect did not interact with target congruency. Thus, older adults engaged similar mechanisms of conflict processing and their cortical systems underlying inhibitory processes were relatively intact. Whereas this study is to the best of our knowledge the first to employ ERPs to examine the ANT in older adults, our young adult data support recent ERP studies of the ANT that have demonstrated P300 reductions to incongruent vs. congruent target stimuli (e.g., Neuhaus et al., 2007, 2010). Similar congruency effects have been observed in Stroop paradigms (Zurrón et al., 2009), which have been interpreted in the context of well-replicated reductions in posterior P300 amplitude as task difficulty increases (Polich, 1987; Katayama and Polich, 1998). Given these consistent findings across different paradigms, it appears that reductions in P300 amplitudes reflect a disrupted allocation of attention that is associated with processing incongruent stimuli relative to congruent stimuli. Importantly, our findings suggest that congruency effects on P300 amplitudes are present in both young and older adults.

Taken together, our findings revealed significant behavioral and neural alterations of the alerting network in older adults. Cues designed to facilitate an alerting response were less effective in older than young adults. While alerting cues enhanced subsequent ERP reflections of attentional processing in younger adults, older adults did not show this effect. In fact, reductions in the N1 alerting effect in older adults help to account for broader age-related neuropsychological declines in attention. By contrast, ERP correlates of orienting and executive control networks reflected generalized amplitude reductions consistent with normal aging. These novel findings reveal that aging does not impact all networks of attention equally, but is uniquely associated with impairments in attentional alerting.

With regard to study limitations, the version of the ANT used in this study did not include an “invalid” spatial cueing condition, which would have enabled us to examine the full complement of engagement *and* disengagement of attentional orienting. Thus, we cannot interpret our results in terms of potential age-related deficits in disengagement (c.f., Shulman et al., 2010). Recently, several investigators have included invalid cues in their ANT paradigms, and have noted both beneficial and detrimental effects on the ability to overcome response conflict following valid and invalid cues, respectively (e.g., Fan et al., 2009). Specifically, whereas valid cues likely enhance prepotent stimulus processing and effective shifting (i.e., orienting) of attention, invalid cueing conditions likely require the re-direction of attentional processes and the use of additional resources for conflict resolution. Consequently, future studies should investigate the differential effects of cue-validity on early ERP components, which have been found to be differentially sensitive to the effects of valid and invalid cueing conditions (e.g., Wright et al., 1995; Talsma et al., 2005). Similarly, the use of vertical, rather than lateralized cueing may have obscured orienting effects, as evidenced by small N1 to the spatial vs. center-cue conditions. Future ERP studies of orienting may benefit from use of lateralized attention network paradigms used in previous behavioral studies (e.g., Greene et al., 2008).

A broader problem associated with the ANT is the use of cognitive subtractions. For example, the alerting effect is typically assumed to reflect a contrast between two similar cognitive states that are differentiated only by cues. However, the condition in which no cue is provided may create additional difficulty for participants, as they do not know which type of stimulus will be presented next (Galvao-Carmona et al., 2014). By contrast, the appearance of the double cue removes uncertainty about the upcoming stimulus, particularly when these cues are always valid. Thus, a differential cognitive load may develop and interact with participant expectations during these trials, rendering a simplistic cognitive subtraction inadequate. In the current study, the additional analysis of ERP correlates in a factorial design produced ANOVA results that supported the findings of the cognitive subtractions, suggesting that the observed alerting effects cannot be dismissed as a simplistic comparison of two conditions.

An additional limitation was that our sample size was relatively small, which may have introduced variability and influenced our ability to detect significant differences. Finally, both groups of participants performed the task with very high accuracy. The near-ceiling performance accuracy precludes our ability to examine task errors on the ANT more fully, given their low frequency and non-normal distribution. Insufficient numbers of trials also hindered our ability to separately examine the neural reflections of each congruency condition as a function of cue condition. Instead, target congruency had to be collapsed across conditions in order to observe the effects of cue, which may have masked potentially meaningful insights about the interactions between cues and subsequent attentional targets.

## Summary and Conclusions

The current study examined age-related differences on the behavioral and neural correlates of three attentional networks (alerting, orienting, and executive control). Results revealed significant behavioral and ERP alterations in the alerting system of healthy older adults, even after controlling for generalized slowing and amplitude reductions. It appears that older adults had selective difficulty engaging the alerting network of attention, which prevented efficient facilitation of attention and subsequent responding. However, there is good news for those feeling “antsy” about age effects on attentional networks, as older adults did not exhibit behavioral or neural differences in orienting and executive control networks. As such, older adults may not be as efficiently alerted to upcoming information, yet they are equally capable in other aspects of attentional function. As the world’s population ages, these results have important implications for our understanding of healthy aging.

## Ethics Statement

The study was approved by the University of Florida, Health Sciences Institutional Review Board.

## Author Contributions

DASK was responsible for experimental design, data collection, and manuscript preparation. CNS was responsible for data collection, data analysis, and manuscript preparation. VMD was responsible for manuscript preparation. WMP was responsible for manuscript preparation.

## Funding

This work was supported by Grants from the NIH (T32-AG020499 to DASK, R21-MH0737076 to WMP, and R21-NS079767).

## Conflict of Interest Statement

The authors declare that the research was conducted in the absence of any commercial or financial relationships that could be construed as a potential conflict of interest.

## References

[B1] Beck A. T. (1996). Beck Depression Inventory - Second Edition (BDI-II). San Antonio, TX, USA: The Psychological Corporation.

[B3] Bush L. Hess U. Wolford G. (1993). Transformations for within-subject designs: a monte carlo investigation. Psychol. Bull. 113, 566–579. 10.1037/0033-2909.113.3.566 8316614

[B4] Cohen J. (1988). Statistical Power Analysis for the Behavioral Sciences. Hillsdale, NJ: Erlbaum Associates.

[B6] Eriksen B. A. Eriksen C. W. (1974). Effects of noise letters upon the identification of a target letter in a nonsearch task. Percept. Psychophys. 16, 143–149. 10.3758/bf03203267

[B8] Fan J. Byrne J. Worden M. S. Guise K. G. McCandliss B. D. Fossella J.. (2007). The relation of brain oscillations to attentional networks. J. Neurosci. 27, 6197–6206. 10.1523/JNEUROSCI.1833-07.2007 17553991 PMC6672149

[B9] Fan J. Gu X. Guise K. G. Liu X. Fossella J. Wang H.. (2009). Testing the behavioral interaction and integration of attentional networks. Brain Cogn. 70, 209–220. 10.1016/j.bandc.2009.02.002 19269079 PMC2674119

[B10] Fan J. Hof P. R. Guise K. G. Fossella J. A. Posner M. I. (2007). The functional integration of the anterior cingulate cortex during conflict processing. Cereb. Cortex 187, 796–805. 10.1093/cercor/bhm125 17652463

[B11] Fan J. McCandliss B. D. Fossella J. Flombaum J. I. Posner M. I. (2005). The activation of attentional networks. Neuroimage 26, 471–479. 10.1016/j.neuroimage.2005.02.004 15907304

[B12] Fan J. McCandliss B. D. Sommers T. Raz A. Posner M. I. (2002). Testing the efficiency and independence of attentional networks. J. Cogn. Neurosci. 14, 340–347. 10.1162/089892902317361886 11970796

[B7] Fan J. Posner M. (2004). Human attentional networks. Psychiatr. Prax. 31, S210–S214. 10.1055/s-2004-828484 15586312

[B14] Faust M. E. Balota D. A. Spieler D. H. Ferraro F. R. (1999). Individual differences in information processing rate and amount: implications for group differences in response latency. Psychol. Bull. 125, 777–799. 10.1037/0033-2909.125.6.777 10589302

[B15] Fernandez-Duque D. Black S. E. (2006). Attentional networks in normal aging and Alzheimer’s disease. Neuropsychology 20, 133–143. 10.1037/0894-4105.20.2.133 16594774

[B16] Fernandez-Duque D. Posner M. I. (1997). Relating the mechanisms of orienting and alerting. Neuropsychologia 35, 477–486. 10.1016/s0028-3932(96)00103-0 9106276

[B17] Fjell A. M. Walhovd K. B. (2001). P300 and neuropsychological tests as measures of aging: scalp topography and cognitive changes. Brain Topogr. 14, 25–40. 10.1023/A:1012563605837 11599530

[B18] Folk C. L. Hoyer W. J. (1992). Aging and shifts of visual spatial attention. Psychol. Aging 7, 453–465. 10.1037/0882-7974.7.3.453 1388867

[B19] Folstein M. F. Folstein S. E. McHugh P. R. (1975). “Mini-mental state”: a practical method for grading the cognitive state of patients for the clinician. J. Psychiatr. Res. 12, 189–198. 10.1016/0022-3956(75)90026-6 1202204

[B20] Ford J. M. Roth W. T. Isaacks B. G. White P. M. Hood S. H. Pfefferbaum A. (1995). Elderly men and women are less responsive to startling noises: N1, P3 and blink evidence. Biol. Psychol. 39, 57–80. 10.1016/0301-0511(94)00959-2 7734630

[B21] Forstmann B. U. Tittgemeyer M. Wagenmakers E. J. Derrfus J. Imperait E. Brown S. (2011). The speed-accuracy tradeoff in the elderly brain: a structural model-based approach. J. Neurosci. 31, 17242–17249. 10.1523/JNEUROSCI.0309-11.2011 22114290 PMC6623864

[B23] Galvao-Carmona A. González-Rosa J. J. Hidalgo-Muñoz A. R. Parámo D. Benétez M. L. Izquierdo G.. (2014). Disentangling the attention network test: behavioral, event related potentials and neural source analysis. Front. Hum. Neurosci. 8:813. 10.3389/fnhum.2014.00813 25352800 PMC4195286

[B230] Gamboz N. Zamarian S. Cavellero C. (2010). Age-related differences in the attention network test (ANT). Exp. Aging Res. 36, 287–305. 10.1080/0361073X.2010.484729 20544449

[B24] Golden C. J. (1978). Stroop Color and Word Test. Chicago: Stoelting.

[B25] Golub E. J. Miranda G. G. Johnson J. K. Starr A. (2001). Sensory cortical interactions in aging, mild cognitive impairment and Alzheimer’s disease. Neurobiol. Aging 22, 755–763. 10.1016/s0197-4580(01)00244-5 11705635

[B26] Greene D. J. Barnea A. Herzberg K. Rassis A. Neta M. Raz A.. (2008). Measuring attention in the hemispheres: the lateralized attention network test (LANT). Brain Cogn. 66, 21–31. 10.1016/j.bandc.2007.05.003 17590491 PMC4283820

[B27] Greenwood P. M. Parasuraman R. Haxby J. V. (1993). Visuospatial attention across the adult lifespan. Neuropsychologia 31, 471–485. 10.1016/0028-3932(93)90061-4 8502379

[B28] Harter M. R. Miller S. L. Price N. J. LaLonde M. E. Keyes A. L. (1989). Neural processes involved in directing attention. J. Cogn. Neurosci. 1, 223–237. 10.1162/jocn.1989.1.3.223 23968506

[B29] Hartley A. A. (1993). Evidence for the selective preservation of spatial selective attention in old age. Psychol. Aging 8, 371–379. 10.1037/0882-7974.8.3.371 8216957

[B30] Hartley A. A. Kieley J. M. Slabach E. H. (1990). Age differences and similarities in the effects of cues and prompts. J. Exp. Psychol. Hum. Percept. Perform. 16, 523–537. 10.1037/0096-1523.16.3.523 2144568

[B31] Hillyard S. A. Vogel E. K. Luck S. J. (1998). Sensory gain control (amplification) as a mechanism of selective attention: electrophysiological and neuroimaging evidence. Philos. Trans. R. Soc. Lond. B Biol. Sci. 353, 1257–1270. 10.1098/rstb.1998.0281 9770220 PMC1692341

[B32] Hopf J. M. Mangun G. R. (2000). Shifting attention in space: an electrophysiological analysis using high spatial resolution mapping. Clin. Neurophysiol. 111, 1241–1257. 10.1016/s1388-2457(00)00313-8 10880800

[B33] Jennings J. M. Dagenbach D. Engle C. M. Funke L. J. (2007). Age-related changes and the attention network task: an examination of alerting, orienting and executive function. Neuropsychol. Dev. Cogn. B Aging Neuropsychol. Cogn. 14, 353–369. 10.1080/13825580600788837 17612813

[B34] Katayama J. Polich J. (1998). Stimulus context determines P3a and P3b. Psychophysiology 35, 23–33. 10.1111/1469-8986.3510023 9499703

[B35] Larson M. J. Kaufman D. A. Perlstein W. M. (2009). Neural time course of conflict adaptation effects on the Stroop task. Neuropsychologia 47, 663–670. 10.1016/j.neuropsychologia.2008.11.013 19071142

[B36] Luck S. J. (2005). An Introduction to the Event-Related Potential Technique Cambridge, MA: MIT Press.

[B37] MacLeod J. W. Lawrence M. A. McConnell M. M. Eskes G. A. Klein R. M. Shore D. I. (2010). Appraising the ANT: psychometric and theoretical considerations of the attention network test. Neuropsychology 24, 637–651. 10.1037/a0019803 20804252

[B38] Mangun G. R. (1995). Neural mechanisms of visual selective attention. Psychophysiology 32, 4–18. 10.1111/j.1469-8986.1995.tb03400.x 7878167

[B39] Neter J. Wasserman W. Kutner M. H. (1985). Applied Linear Statistical Models: Regression, Analysis of Variance and Experimental Designs. 2nd Edn. Homewood, IL: R.D. Irwin.

[B40] Neuhaus A. H. Koehler S. Opgen-Rhein C. Urbanek C. Hahn E. Dettling M. (2007). Selective anterior cingulate cortex deficit during conflict solution in schizophrenia: an event-related potential study. J. Psychiatr. Res. 41, 635–644. 10.1016/j.jpsychires.2006.06.012 16908030

[B41] Neuhaus A. H. Urbanek C. Opgen-Rhein C. Hahn E. Ta T. M. T. Koehler S.. (2010). Event-related potentials associated with Attention Network Test. Int. J. Psychophysiol. 76, 72–79. 10.1016/j.ijpsycho.2010.02.005 20184924

[B42] Nobre A. C. Sebestyen G. N. Miniussi C. (2000). The dynamics of shifting visuospatial attention revealed by event-related potentials. Neuropsychologia 38, 964–974. 10.1016/s0028-3932(00)00015-4 10775707

[B43] Polich J. (1987). Task difficulty, probability and inter-stimulus interval as determinants of P300 from auditory stimuli. Electroencephalogr. Clin. Neurophysiol. 68, 311–320. 10.1016/0168-5597(87)90052-9 2439311

[B44] Polich J. (2007). Updating P300: an integrative theory of P3a and P3b. Clin. Neurophysiol. 118, 2128–2148. 10.1016/j.clinph.2007.04.019 17573239 PMC2715154

[B45] Posner M. I. (1980). Orienting of attention. Q. J. Exp. Psychol. 32, 3–25. 7367577 10.1080/00335558008248231

[B46] Posner M. I. Fan J. (2004). “Attention as an organ system,” in Topics in Integrative Neuroscience: From Cells to Cognition, eds Pomerantz J. R. Crair M. C. (Cambridge: Cambridge University Press), 31–61.

[B47] Posner M. I. Peterson S. E. (1990). The attention system of the human brain. Annu. Rev. Neurosci. 13, 25–42. 10.1146/annurev.neuro.13.1.25 2183676

[B48] Posner M. I. Sheese B. E. Odludaş Y. Tang Y. (2006). Analyzing and shaping human attentional networks. Neural Netw. 19, 1422–1429. 10.1016/j.neunet.2006.08.004 17059879

[B49] Ratcliff R. (1993). Methods for dealing with reaction time outliers. Psychol. Bull. 114, 510–532. 10.1037/0033-2909.114.3.510 8272468

[B51] Reitan R. M. Wolfson D. (1995). Category test and trail making test as measures of frontal lobe functions. Clin. Neuropsychol. 9, 50–56. 10.1080/13854049508402057

[B54] Scherg M. (1990). “Fundamentals of dipole source potential analysis,” in Auditory Evoked Magnetic Fields and Electric Potentials. Advances in Audiology, (Vol. 6) eds Grandori F. Hoke M. (Basel: Karger), 65–78.

[B55] Shulman G. L. Pope D. L. Astafiev S. V. McAvoy M. P. Snyder A. Z. Corbetta M. (2010). Right hemisphere dominance during spatial selective attention and target detection occurs outside the dorsal frontoparietal network. J. Neurosci. 30, 3640–3651. 10.1523/JNEUROSCI.4085-09.2010 20219998 PMC2872555

[B56] Speilberger C. D. Gorusch R. L. Lushene R. Vagg P. R. Jacobs G. A. (1983). Manual for the State-Trait Anxiety Inventory. Palo Alto, CA: Consulting Psychologists Press.

[B57] Starkstein S. E. Mayberg H. S. Preziosi T. J. Andrezejewski P. Leiguarda R. Robinson R. G. (1992). Reliability, validity and clinical correlates of apathy in Parkinson’s disease. J. Neuropsychiatry Clin. Neurosci. 4, 134–139. 10.1176/jnp.4.2.134 1627973

[B58] Talsma D. Slagter H. A. Nieuwenhuis S. Hage J. Kok A. (2005). The orienting of visuospatial attention: an event-related potential study. Brain Res. Cogn. Brain Res. 25, 117–129. 10.1016/j.cogbrainres.2005.04.013 15925498

[B59] van Veen V. Carter C. S. (2002). The anterior cingulate as a conflict monitor: fMRI and ERP studies. Physiol. Behav. 77, 477–482. 10.1016/s0031-9384(02)00930-7 12526986

[B61] Verhaeghen P. Cerella J. (2002). Aging, executive control and attention: a review of meta-analyses. Neurosci. Biobehav. Rev. 26, 849–857. 10.1016/s0149-7634(02)00071-4 12470697

[B60] Verhaeghen P. De Meersman L. (1998). Aging and the Stroop effect: a meta-analysis. Psychol. Aging 13, 120–126. 10.1037/0882-7974.13.1.120 9533194

[B62] Wechsler D. A. (1997). Wechsler Adult Intelligence Scale. 3rd Edn. San Antonio, TX: The Psychological Corporation.

[B64] Wolfinger R. Tobias R. Sall J. (1994). Computing gaussian likelihoods and their derivatives for general linear mixed models. SIAM J. Sci. Comput. 15, 1294–1310. 10.1137/0915079

[B65] Wright M. J. Geffen G. M. Geffen L. B. (1995). Event-related potentials during covert orientation of visual-attention: effects of cue validity and directionality. Biol. Psychol. 41, 183–202. 10.1016/0301-0511(95)05128-7 8534791

[B66] Yesavage J. A. Brink T. L. Rose T. L. Lum O. Huang V. Adey M.. (1983). Development and validation of a geriatric depression screening scale: a preliminary report. J. Psychiatr. Res. 17, 37–49. 10.1016/0022-3956(82)90033-4 7183759

[B660] Zhou S. S. Fan J. Lee T. M. Wang C. Q. Wang K. (2011). Age-related differences in attentional networks of alerting and executive control in young, middle-aged, and older Chinese adults. Brain Cogn. 75, 205–210. 10.1016/j.bandc.2010.12.003 21251744

[B67] Zurrón M. Pousoa M. Lindína M. Galdoa S. Díaza F. (2009). Event-related potentials with the Stroop colour-word task: timing of semantic conflict. Int. J. Psychophysiol. 72, 246–252. 10.1016/j.ijpsycho.2009.01.002 19171167

